# Acute oral toxicity and biodistribution study of zinc-aluminium-levodopa nanocomposite

**DOI:** 10.1186/s11671-015-0742-5

**Published:** 2015-03-01

**Authors:** Aminu Umar Kura, Bullo Saifullah, Pike-See Cheah, Mohd Zobir Hussein, Norazrina Azmi, Sharida Fakurazi

**Affiliations:** Laboratory of Vaccines and Immunotherapeutics, Institute of Bioscience, Universiti Putra Malaysia, 43400 Selangor, Malaysia; Materials Synthesis and Characterization Laboratory, Institute of Advanced Technology, Universiti Putra Malaysia, 43400 UPM Serdang, Selangor, Malaysia; Neurobiology and Genetic Group, Genetic Medicine Research Centre, Faculty of Medicine and Health Science, Universiti Putra Malaysia, 43400 Serdang, Selangor, Malaysia; Department of Human Anatomy, Faculty of Medicine and Health Sciences, Universiti Putra Malaysia, 43400 Serdang, Selangor, Malaysia; Faculty of Pharmacy, Universiti Kebangsan Malaysia, Jalan Raja Muda Abdul Aziz, 50300 Kuala Lampur, Malaysia

**Keywords:** Acute toxicity, Nanocomposite, Layered hydroxide, Levodopa

## Abstract

Layered double hydroxide (LDH) is an inorganic–organic nano-layered material that harbours drug between its two-layered sheets, forming a sandwich-like structure. It is attracting a great deal of attention as an alternative drug delivery (nanodelivery) system in the field of pharmacology due to their relative low toxic potential. The production of these nanodelivery systems, aimed at improving human health through decrease toxicity, targeted delivery of the active compound to areas of interest with sustained release ability. In this study, we administered zinc-aluminium-LDH-levodopa nanocomposite (ZAL) and zinc-aluminium nanocomposite (ZA) to Sprague Dawley rats to evaluate for acute oral toxicity following OECD guidelines. The oral administration of ZAL and ZA at a limit dose of 2,000 mg/kg produced neither mortality nor acute toxic signs throughout 14 days of the observation. The percentage of body weight gain of the animals showed no significant difference between control and treatment groups. Animal from the two treated groups gained weight continuously over the study period, which was shown to be significantly higher than the weight at the beginning of the study (*P* < 0.05). Biochemical analysis of animal serum showed no significant difference between rats treated with ZAL, ZA and controls. There was no gross lesion or histopathological changes observed in vital organs of the rats. The results suggested that ZAL and ZA at 2,000 mg/kg body weight in rats do not induce acute toxicity in the animals. Elemental analysis of tissues of treated animals demonstrated the wider distribution of the nanocomposite including the brain. In summary, findings of acute toxicity tests in this study suggest that zinc-aluminium nanocomposite intercalated with and the un-intercalated were safe when administered orally in animal models for short periods of time. It also highlighted the potential distribution ability of Tween-80 coated nanocomposite after oral administration.

## Background

Levodopa is still the gold standard in symptomatic treatment of Parkinson’s disease (PD), though there are many therapeutic options and the list kept on expanding tremendously, all with the view to improve on PD management [1]. A major therapeutic challenge and controversy in the usage of levodopa is its potential to cause toxic effects on nigrostriatal cells. This will potentiate the progression of neurodegeneration and ultimately worsening of Parkinson’s disease symptoms [1]. There is no direct evidence showing levodopa causing toxicity in PD patient, but there are increasing cell and animal study evidence of such [1]. This is adding to the panic and worries of possible neurotoxicity in human being [1]. Levodopa has the ability to potentiate free radical productions [1], which are known to be toxic to cells. Another setback with levodopa usage is its potential to cause long-term adverse effects of dyskinesia and other motor fluctuations. These side effects were believed to be due to pulsatile stimulation of the dopaminergic neuron at the substantia nigra by levodopa leading to ‘off and on’ excitation of the neurons [1].

Layered double hydroxide (LDH) compounds of magnesium and aluminium ions are attracting a great deal of attention, as alternative drug delivery system in the field of pharmacology [2-4]. This is due to their low toxicity potential compared to the other nanodelivery systems [2,5]. The inorganic nano-layered material harbours drug between two layered sheets, forming a sandwich-like structure [3]. Drug release by this noble carrier follows the principle of ion exchange that governs drug intercalation during synthesis, especially in lower acidic environment, while burst release is seen in an acidic environment [2,3]. Surrounding anions such as Cl^−^ and/or phosphate exchanges for the anionic drug intercalated between the nanosheets [4,6]. Treatment of peptic ulcer diseases using LDH nano-carrier as oral antacids and anti-pepsin is a clear attestation to the established safety and biocompatibility of this nanodelivery system [7]. Among the different types of inorganic nanodelivery systems, LDH of either aluminium or magnesium is shown to be the least toxic [5], making it a widely used alternative drug delivery system containing different type of drugs, genes and proteins [3,4].

Nanodelivery systems are generally aimed at improving human health through decreased toxicity, targeted delivery of the active compound to areas of interest and sustained release. Nevertheless, of concern is the toxicity of some of the nanodelivery systems, which could be from the material used, the synthetic process, the drug intercalated, the coating agent, the particle size, the distribution pattern and the end products of the delivery system [3,8,9]. Thus, comprehensive assessment of any newly synthesized nanomaterial is inevitable, although *in vivo* tests are supposed to be replaced by *in vitro* assessment due to time-consuming, cost and related ethical issues. However, *in vivo* toxicity assessment is still recommended due to the complexity of the body system, which may influence the pharmacokinetics of nanodelivery system, hitherto not seen during an *in vitro* toxicity tests, and vice versa. This will give valuable inputs in the real-life system.

Pharmaceutical formulations including nanodelivery systems (NDSs) are taken into the human body through any of the several routes of drug administration [10,11]. The route of exposure may influence the distribution and bioavailability, especially transport across biological barriers like the brain [11]. Oral route in drug administration is perhaps the most preferred by the patient and the clinician alike; it is the most convenient, cost effective and relatively easier to use [12]. Thus, it has the highest level of patient compliance, although it is not without some limitation. Among the various limitations of oral route for drug delivery are hepatic first pass metabolism and enzymatic degradation within the gastrointestinal tract [13]. These factors are prohibiting oral administration of certain classes of drugs, especially proteins and the peptides [14]. Consequently, parenteral routes like intravenous, intramuscular and subcutaneous routes of administration are resorted to deliver some agents [2,3]. Nanotechnology base-drug delivery systems are now offering many advantages over the conventional system. They have good drug intracellular penetration and enhanced absorption into targeted tissues, even where orally administered. They also show better pharmacokinetic (PK) properties, increased clinical efficacy and reduced toxicity in most cases [15].

Surface coating of nanodelivery systems with linkers like chitosan, polyethylene glycol (PEG), polysorbate-80 (Tween-80), polyvinyl alcohol (PVA) or polysaccharide can prevent aggregation, enhance solubility, minimize nonspecific binding, prolong circulation time and at times serve as a specific tissue-targeting mechanism [16]. Thus, given a nanodelivery system, better distribution increases serum half-life and wider application both in parenteral and nonparenteral administration.

The presence of the blood–brain barrier (BBB) is affecting treatment of brain pathologies with the conventional and most nanodelivery systems. The barrier excludes compounds from reaching the brain in the desired concentration, thus limiting the expected therapeutic effect in many central nervous system (CNS) diseases [17]. A very promising approach in overcoming this obstacle is the use of the nanodelivery system especially after surface modification with polymers or surfactants [18]. Tween-80 coating of nanoparticle was reported to be helpful in delivering drugs to the brain [19,20]. Materials coated with this surfactant were capable of transporting loaded drugs across the BBB after oral and intravenous administration. Apolipo-protein-B acquisition on the surface of this material was shown to be the mediator behind this success, where the acquired protein is transported via LDH receptors located at the endothelium of the BBB [19]. Then, the active drug loaded on the nanodelivery system will be released inside the brain.

Nanomaterial’s size is also crucial in determining nanoparticle distribution; the reticule endothelial systems, notably in the liver, spleen and kidney, were implicated in sequestrating larger percentage of these materials within the range of 100 to 250 nm [21]. Nevertheless, surface modification was reported to alter these facts; for example, a layered double hydroxide nanocomposite with diameter size of 100 to 200 nm coated with chitosan showed a preference in distribution to the liver, lung, spleen and kidney [22]. Delivery of the same nanocomposite to the lung, but not the liver, kidney or spleen, was achieved through further addition of more chitosan [22]. This preferential distribution of the nanomaterial following addition of coating substance is attributed to better solubility, lesser aggregation and improved dispersion.

Our previous studies provided detail synthesis, surface coating and characterization of zinc-aluminium-levodopa nanocomposite (ZAL), a nanodelivery system for the treatment of Parkinson’s disease [3,23]. Briefly, zinc and aluminium molar ratio of 2:1 were used via co-precipitation in the synthesis. The successful intercalation of levodopa was demonstrated using X-ray diffraction and Fourier transform infrared spectroscopy (FTIR), in which the new delivery system contains about 16% levodopa by weight. It was shown to gain added thermal stability and exhibited a sustained, continuous and controlled levodopa release from the two pH value tested [3]. Toxicity result using the nanodelivery technique on cell line in the same study was demonstrated to be reduced compared to pure levodopa. Further coating of ZAL with polysorbate-80 (Tween-80) showed an improved release profile and a better cell viability ([23]. Repeated dose of the same nanocomposite in animal study showed slight liver and renal derangement [10]. However, establishing the entire toxicological profile using an animal model, acute and chronic toxicity assessment is also indispensable.

In this study, the acute oral toxicity and distribution potentials of Tween-80-coated zinc-aluminium-levodopa nanocomposite were evaluated according to the fixed dose procedures (OECD, 2002) [24]. Changes in coefficients of tissues to body weight, gross and histopathological deviations, as well as alteration in biochemical parameters such as liver and renal enzymes, were assessed. Synthesis of ZAL was aimed to provide an alternative to Parkinson’s disease treatment strategy. Thus, providing complete information on the safety of this layered hydroxide nanocomposite in drug delivery is the primary objective of this study.

## Methods

### Animals

Male Sprague Dawley rats were 6 to 8 weeks old; average weight of 200 ± 20 g were obtained from in-house animal facility and used as experimental animals. The rats were housed in plastic cages, three rats per cage, and maintained in the animal house of the Department of Human Anatomy, Faculty of Medicine and Health Sciences Universiti Putra Malaysia. They were maintained under standard conditions of temperature 25°C ± 2°C, relative humidity 70% ± 5% and 12-h light–dark cycle. Feeding of the animals and water intake were done with standard rat pellets and tap water *ad libitum* throughout the experiments. In general, animal handling, from the beginning to the end of the study, was ethically done according to the agreed guidelines for the university, Universiti Putra Malaysia Institutional Animal Care and Use Committee (IACUC) [25].

### Acute oral toxicity test in rats

Animals were kept for 5 days to allow for acclimatization to the laboratory conditions before commencement of the study. A single (1 day) oral dose of the drugs was given as per the Organization for Economic Co-operation and Development (OECD) 407 guidelines (OECD, 2002) [12]. Twenty-one animals were distributed randomly into three groups (Table 1): group 1, zinc-aluminium-levodopa nanocomposite (ZAL 2,000 mg/kg); group 2, zinc-aluminium nanocomposite (ZA 2,000 mg/kg); group 3, vehicle control (normal saline 100 mL/kg) body weight (Table 1).

**Table 1:** **Arrangement of rats into different groups**

**Groups**	**Dose** ** (** **concentration** **)**	**Number of rats**
ZAL	2,000 mg/kg	7
ZA	2,000 mg/kg	7
VC	100 mL/kg (body weight)	7

A table showing grouping arrangement and doses of nanocomposite given to animals (*n* = 7) ZAL (zinc-aluminium-levodopa nanocomposite), ZA (zinc-aluminium nanocomposite) and VC (vehicle control).

A freshly prepared nanocomposite was given to each rat in treatment groups, while the rats in the control group received only normal saline. At the beginning of the study (marked as day 0), the weight of animals was recorded, thereafter the body weight was recorded at day 7 and lastly on day 14 just before the sacrifice. Animals were monitored at intervals of 30 min, 1 h, 2 h, 4 h and 12 h on the first day and subsequently twice daily during the course of treatment, observing for any clinical signs of toxicity and possible mortality. At the end of 14 days of observation, animals were sacrificed via exsanguination through cardiac puncture following anaesthesia with ketamine and xylazine at a dose of 80 and 10 mg/kg, respectively. The brain, liver, spleen, heart and kidney were harvested, weighed and macroscopically examined for lesions and/or abnormalities.

### Coefficients of the brain, liver, spleen, heart and kidney

The coefficients of these organs, the brain, liver, spleen, heart and kidney, were calculated as the ratio of the organs to body weight [the ratio of organ (wet weight, mg) to body weight (g)].

### Biochemical analysis

Blood taken was placed in a plain 2 mL Eppendorf tube and allowed to stand for about 30 min before it was centrifuged at 1,500 rpm for 5 min (Eppendorf 5810R, Hamburg, Germany), at room temperature; the serum obtained was used for urea, sodium, potassium, creatinine, chloride, AST, ALT and GGT using a diagnostic kits (Roche, Basel, Switzerland) in an automatic biochemistry analyser (Hitachi 902, Tokyo, Japan).

### Histopathological evaluation

Postmortem examination was conducted immediately after sacrifice to detect the presence of any abnormality on the animal’s vital organs. The gross appearance of these organs was observed with the naked eye, while the histological appearance of the liver, spleen, kidney and brain were observed after haematoxylin-eosin (H&E) stain. The animals were subjected to trans-cardiac perfusion using 4% paraformaldehyde after anaesthesia. The tissues were later subjected to standard tissue processing and were embedded in paraffin blocks. The tissues were microsectioned into 5 μm thickness and mounted onto glass slides. The H&E staining technique was used to stain the slides, and they were viewed using an optical microscope (Olympus FSX-100, Olympus Corporation, Shinjiku-ku, Tokyo, Japan).

### Nanocomposite size, particle distribution and tissue distribution

The particle size distribution was determined for the levodopa-loaded and unloaded nanocomposite before and after coating with Tween-80. This is to compare and to assess the impact of Tween-80 coating on the nanocomposite. The sizes were obtained by measuring the diameters of about 80 nanocomposites randomly through the TEM images, and the results were analysed using UTHSCSA Image Tool software.

The distribution analysis of Tween-80-coated zinc-aluminium-levodopa nanocomposite was studied from the tissues obtained from rats treated with nanocomposite and control groups. The tissues were obtained from the above-mentioned animal groups as well from our previous sub-acute toxicity study of the same compounds (Table 2) [10]. Tissues of the liver, spleen, kidney and brain were taken out and thawed. From each organ, about 0.3 g of the tissue were collected, weighed, digested and analysed for zinc. Tissue digestion was done as previously described for titanium analysis from mice organs [5]. In brief, equal amount (about 0.3 g) of tissues from each of the above-mentioned organs and 500 μl from serum were digested in a mixture of nitric acid (ultrapure grade) and H_2_O_2_ at ratio 6:1. The mixture was heated at about 160°C using high-pressure reaction container in an oven chamber until the samples were completely digested. Then, the high-pressure containers were let loose and the remaining solution heated at 120°C until the solution was colourless and the remaining nitric acid was removed. The solutions were finally diluted to 10 mL with ultrapure water. Atomic absorption spectroscopy (AAS, Thermo Elemental X7, Thermo Electron Co., Waltham, USA) was used to analyse the zinc concentration in the samples.

**Table 2:** **Animal grouping for distribution study**

**Groups**	**Dose** **(** **concentration** **)**	**Number of rats**
ZAL	2,000 mg/kg (single dose)	7
	500 mg/kg (repeated doses)	7
	5 mg/kg (repeated doses)	7
VC	100 mL/kg (body weight)	7

A table showing grouping arrangement and doses of nanocomposite given to animals (*n* = 7) ZAL (zinc-aluminium-levodopa nanocomposite), ZA (zinc-aluminium nanocomposite) and VC (vehicle control).

### Statistical analysis

Statistical analysis of the obtained data was done using SPSS version 20.0. The mean values and the standard deviations (SDs) of each group were calculated. One-way ANOVA statistical test was used to compare the groups and Turkey or Dunnet’s *post hoc* test was used to compare differences between and within groups when found.

## Results

The figure above shows particle size distribution, mean sizes and shapes of ZA (zinc-aluminium layered double hydroxide nanocomposite coated with Tween-80), ZA-LDH (zinc-aluminium layered double hydroxide nanocomposite), ZAL-LDH (zinc-aluminium-levodopa layered double hydroxide nanocomposite) and ZAL (zinc-aluminium-levodopa layered double hydroxide nanocomposite coated with Tween-80).

### Morbidity and mortality

**Figure 1:**
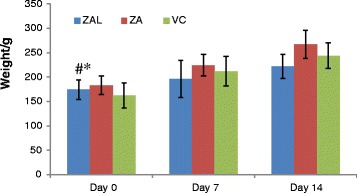
**Bar chart showing an obvious increase in the animals’** **weight in all experimental groups.** There is a statistically significant difference found between day 0 and 14 (#) and day 7 and 14 (*) (*P* < 0.05) in all the groups. One-way ANOVA was used and data are expressed as mean ± SD. Average weight of rats per group at day 0, 7 and 14.

An acute toxicity study using Sprague Dawley was done with a single oral dose of ZAL and ZA of 2,000 mg/kg. The control group (VC) received only normal saline (100 mL/kg) body weight, each group containing seven rats. No mortality was recorded in any of the groups up to day 14 of observations. Behavioural toxicity signs such as convulsion, abnormal posture, paralysis, bleeding, vomiting, diarrhoea, breathing difficulties, restlessness and irritation were also monitored. However, none of the above-mentioned toxicity signs was observed in either the treatment or control groups throughout the 14-day observational period (Table 3). Animals from treatment and control group continued their regular food and water intake accompanied by weight gain over the course of the study (Figure 1). The obvious increase in the animals’ weight was demonstrated to be significantly different between days 0 and 14 and days 7 and 14 with *P* value of <0.05 as tested by one-way ANOVA. When compared between the treated and control groups, the body weight changes were insignificant (Figure 1).

**Table 3:** **Morbidity and mortality data of experimental rats**

**Groups**	**Dose** **(** **concentration** **)**	**Toxicity sign t** **/** **n**	**Mortality d** **/** **a**	**Gross pathology l** **/** **nl**
ZAL	2,000 mg/kg	0/7	0/7	0/7
ZA	2,000 mg/kg	0/7	0/7	0/7
VC	100 mL/kg	0/7	0/7	0/7

Table showing morbidity and mortality data after a single high dose (2,000 mg/kg) given to the rats in the treatment group as well as normal saline given to the control group. Based on the dose used, no rats showed any sign of clinical toxicity, no death recorded and no obvious gross pathology seen in the organs studied. Data are expressed as mean ± SD, *n* = 7. *t/n* toxic/normal, *d/a* dead/alive, *l/nl* lesion/no lesion.

### Coefficients of the brain, liver, spleen, heart and kidney

Organ weight in relation to total body weight (coefficient of organs) of the brain, liver, spleen, heart and kidney from the three groups was also calculated after sacrifice (Table 4). The weights were expressed as milligrams (wet weight of the organs in mg) to (body weight (g)]. There are no significant changes in the coefficients of the brain, liver, spleen, heart and kidney between control and the other two treatment groups (*P* > 0.05).

**Table 4:** **Coefficients of the brain**, **liver**, **spleen**, **heart and right kidneys after a single-dose oral exposure**

**Group**	**Brain mg** **/** **g**	**Liver mg** **/** **g**	**Heart mg** **/** **g**	**Spleen mg** **/** **g**	**Kidney mg** **/** **g**
ZAL	7.85 ± 0.32	39.13 ± 0.17	3.94 ± 0.09	2.66 ± 0.09	5.22 ± 0.12
ZA	6.13 ± 0.29	33.90 ± 2.46	3.81 ± 0.32	2.43 ± 0.15	5.60 ± 0.33
VC	6.94 ± 0.30	36.91 ± 1.77	3.75 ± 0.16	2.82 ± 0.19	4.58 ± 0.34

The table shows the mean coefficient of the brain, liver, spleen, heart and right kidneys of all the groups. To compare the means of each group against the control group, one-way ANOVA was used and it shows no significant difference with *P* > 0.05.

### Biochemical analysis

Table 5 shows the results of biochemical parameters obtained from the rat serum on day 14, post treatment with nanocomposites. The enzymes, ALT, AST, ALP and GGT, were evaluated to ensure the liver functionality. The AST level of ZAL-treated group was shown to be slightly higher than VC and ZA groups. The average value for AST in ZAL-treated group was 152 ± 30 U/L compared to 111 ± 18 and 114 ± 11 U/L from ZA and VC, respectively. Assessment of kidney function was done through checking changes in urea, the level of electrolyte (Na^+^, K^+^ and Cl^−^) and creatinine, where values of control were compared with the two treated groups. Additionally, changes in creatine kinase between control and nanocomposite administered groups were also assessed. However, the difference in the level observed in the liver, kidney and cardiac enzymes of the two treated groups were not significantly different to that of control animals analysed by one-way ANOVA (*P* > 0.05).

**Table 5:** **Changes in biochemical parameters from serum of orally treated rats with nanocomposite**

**Group**	**ALT U** **/** **L**	**ALP U** **/** **L**	**AST U** **/** **L**	**CK U** **/** **L**	**ALT** **/** **AST ratio**	**Creat μmol** **/** **L**	**GGT**	**Urea Mmol** **/** **L**	**Na Mmol** **/** **L**	**K Mmol** **/** **L**	**Cl Mmol** **/** **L**
ZAL	66 ± 17	256 ± 18	152 ± 30	3,889 ± 71	0.43 ± 0.03	48 ± 1	<3	7 ± 1	142 ± 4	9 ± 1	102 ± 3
ZA	61 ± 6	293 ± 38	111 ± 18	3,398 ± 187	0.55 ± 0.06	47 ± 8	<3	6 ± 1	145 ± 12	8 ± 1	103 ± 10
VC	59 ± 8	312 ± 40	114 ± 11	4,410 ± 364	0.51 ± 0.02	46 ± 4	<3	6	143 ± 2	8 ± 1	101 ± 1

All data are expressed as means ± SD, and groups were compared using one-way ANOVA (*n* = 5) for any statistical difference between and within groups. Differences with *P* < 0.05 are considered statistically significant, but none of the parameters measured were found to be significantly different compared to the control group (*P* > 0.05). ALT, alanine aminotransferase; AST, aspartate aminotransferase; CK, creatine kinase; Creat, creatinine; GGT, gamma-glutamyltransferase; Na, sodium; K, potassium; Cl, chloride.

Microscopic appearance of liver stained with H&E.

### Histopathological evaluation

**Figure 2:**
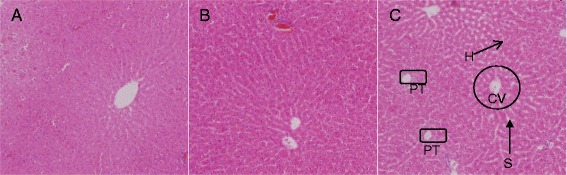
**Histology of the liver tissue after H&E stain in rats.** Histology of the liver tissue after H&E stain (10×) in rats 2/weeks post treatment with ZAL **(A)**, ZA **(B)** and vehicle control **(VC)** single dose each, via the oral route. PT, portal triad; CV, central vein; H, hepatocytes; and S, sinusoid. The hepatic lobular array is well maintained with central vein at the centre shown in the control **(C)**. The same feature was also seen in **A** and **B**. Microscopic appearance of spleen stained with H&E.

**Figure 3:**
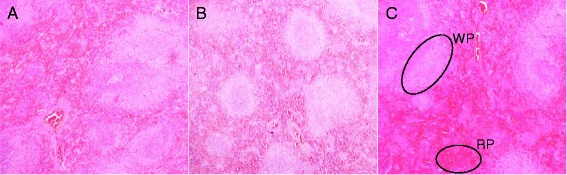
**Histology of the spleen tissue in rats.** Histology of the spleen tissue (10×) in rats 2/weeks post treatment with ZAL **(A)**, ZA **(B)** and vehicle control (VC) single dose each through the oral route. The red pulp (RP) and white pulp (WP) containing lymphocytes where clearly delineated in **C** (control) following H&E stain. Both groups treated with ZAL **(A)** and with ZA **(B)** showed similar morphology as seen in the control **(C)**. Microscopic appearance of kidney stained with H&E.

**Figure 4:**
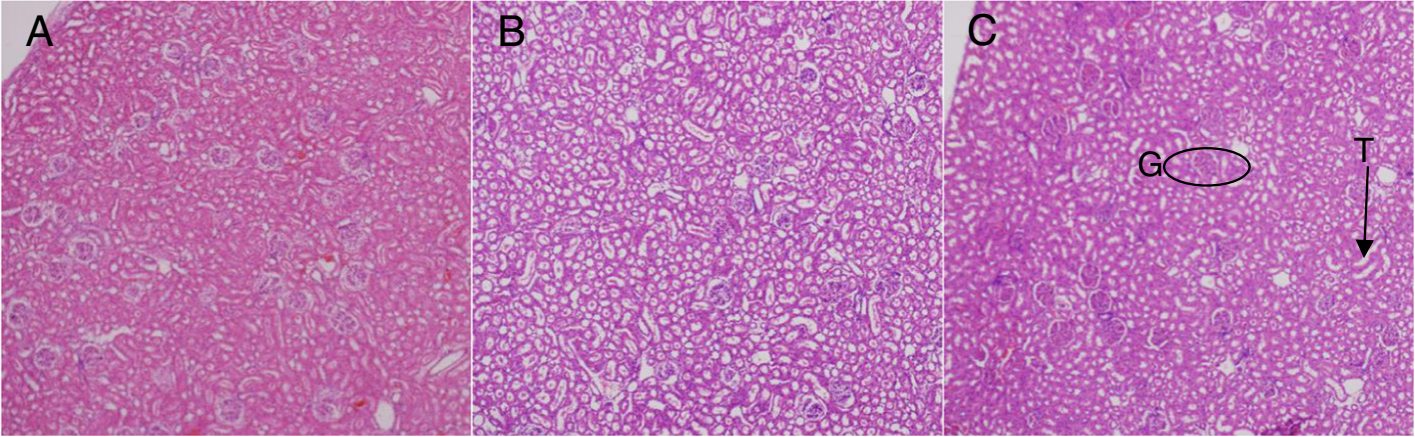
**Histopathology of the kidney tissue** **(** **10** **×)** **2** **/** **weeks post**-**exposure to ZAL (A)** **,** **ZA (B) and vehicle control (C)** **.** G, glomerulus; T, renal tubule. The kidney tissue was stained with H&E, showing preserved glomerular and tubular structure in A and B, similar to the finding in the control group **(C)**. Microscopic appearance of brain cortical region after H&E stain.

**Figure 5:**
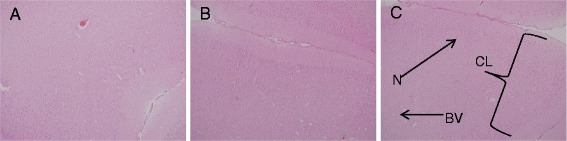
**Histopathology of the brain cortex in rats.** Histopathology of the brain cortex (10×) in rats 2/weeks post-exposure to levodopa nanocomposite and nanocomposite by single oral administration. ZAL **(A)**, ZA **(B)** and vehicle control **(C)**. The cortical layers (CL), neuronal cells (N) and blood vessel (BV) are well delineated in the control group. No obvious changes were noted in the treated groups compared to the control group. Microscopic appearance of striatum stained with H&E.

**Figure 6:**
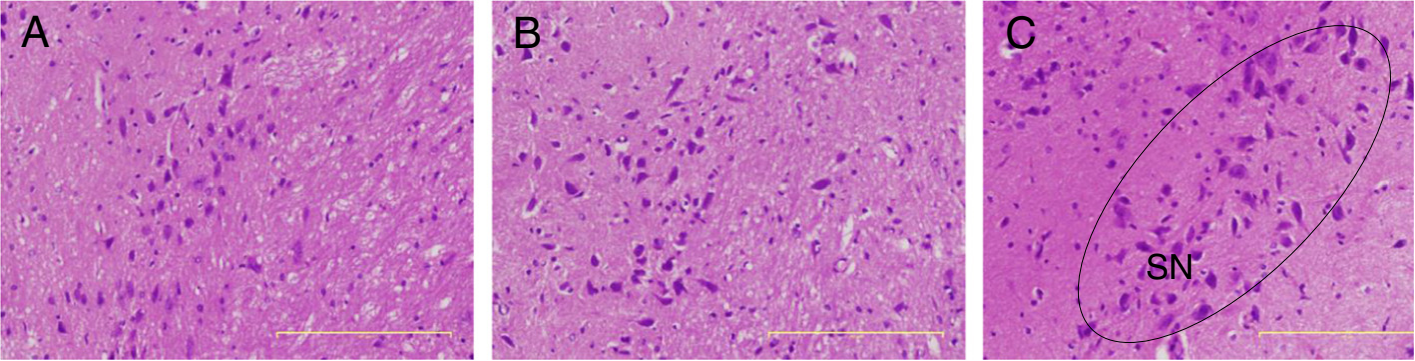
**Histopathology of the striatum in rats.** Histopathology of the striatum (10×) in rats 2/weeks post-exposure to ZAL **(A)**, ZA **(B)** and vehicle control **(C)**. The morphology of the substantia nigra (SN) is well outlined from the brain of the control group **(C)**, similar preservation of the SN structure shown in the two treated groups **(A and B)**.

Histological examination of the liver, spleen, kidney and brain stained with H&E stain in the control and treated animals showed no remarkable lesions that could be attributed to the effect of a single dose of ZAL or ZA (Figures 2, 3, 4, 5 and 6). Hepatic lobular array from the treated group and control was well maintained, central vein (CV) located at the centre surrounded by portal triad (PT) and drained by sinusoids (*S*) (Figure 2). The splenic architecture of the high-dose treated groups showed well-maintained capsule, clearly demarcated white and red pulp and with adjoining trabecular all over the tissues. These histologic features were similar to those of the vehicle control group (Figure 3). The histology of the kidneys showed no pathologic changes in the treated groups when compared to control (Figure 4). The cerebral cortical layers were well delineated in the brain of vehicle-administered group Figure 5C, so is the substantia nigral region in Figure 6C. Findings in the brain of ZAL- and ZA-treated group were remarkably similar to those in the control group (Figures 5A, B and 6A, B). Oral administration of 2,000 mg/kg of layered hydroxide nanocomposite with and without levodopa does not alter the microscopic structure of the vital organs.

### The shape, size and size distribution of particles by TEM analysis

**Figure 7:**
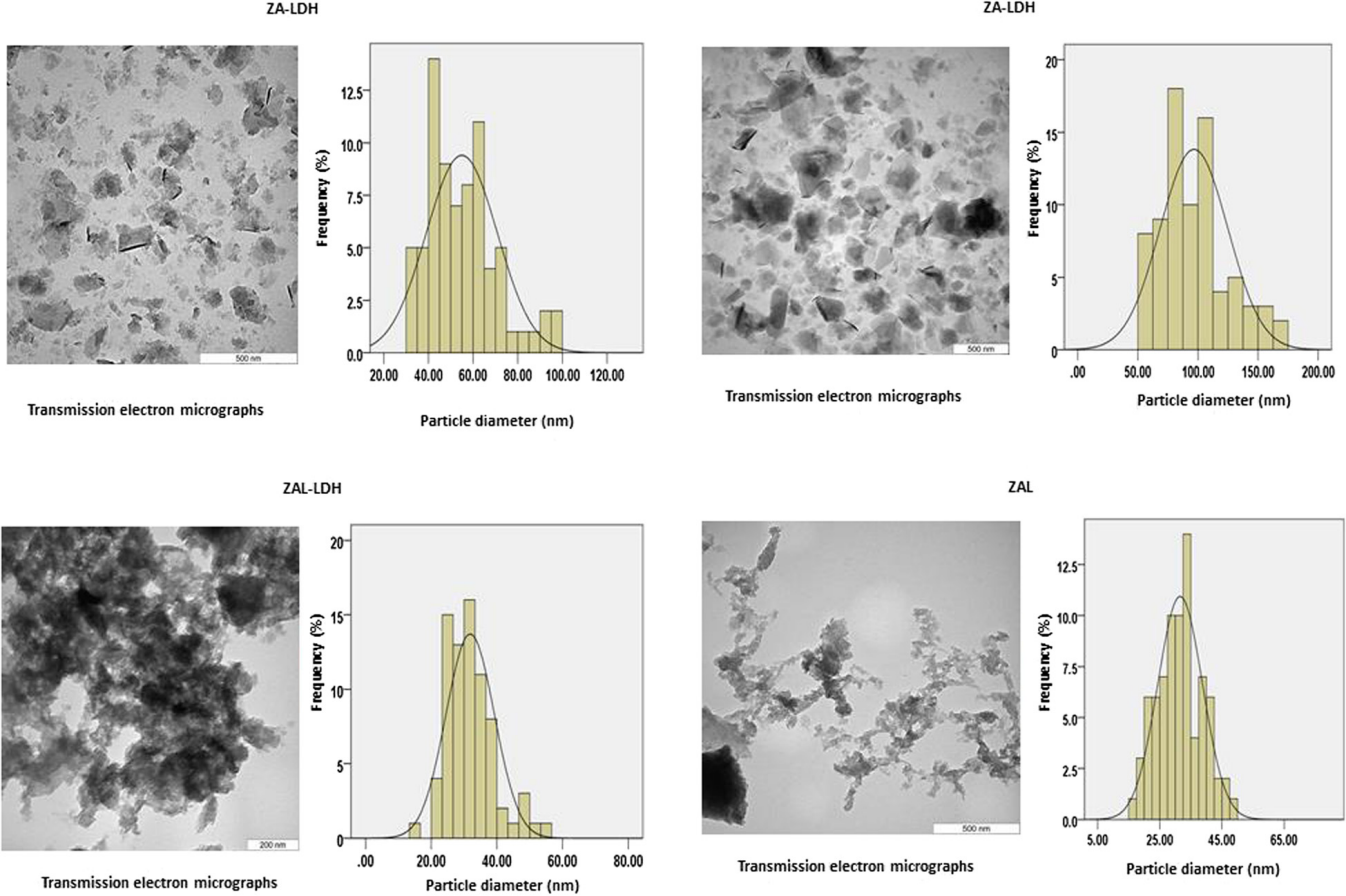
**TEM images of the nanocomposite before and after intercalation with levodopa.**

Transmission electron microscopy (TEM) was used for the determination of shape, size and uniformity of the zinc-aluminium layered double hydroxide nanocomposite (ZA-LDH), Tween-80-coated zinc-aluminium-levodopa layered double hydroxide nanocomposite (ZAL), Tween-80-coated zinc-aluminium layered double hydroxide nanocomposite (ZA) and zinc-aluminium-levodopa layered double hydroxide nanocomposite (ZAL-LDH). The image analysis software was used for the determination of size and the size distribution of all the samples, about 80 particles were randomly chosen from a TEM image of each individual sample. The average size of ZA-LDH was found to be about 55 nm with narrow size distribution and very visible layered shape. The levodopa intercalated nanocomposite ZAL-LDH had agglomerated, particulate type layered shape, with an average size of about 32 nm with narrow size distribution. The ZA, which is LDH coated with Tween-80, had a size of about 97 nm with narrow particle size distribution, and layered shape of Zn/Al-LDHs was retained after Tween-80 coating. The average size for the sample ZAL was determined to be about 31 nm with narrow size distribution and very particulate type layered shape. Figure 7 shows the TEM micrographs and particle size distribution of all of these samples. Both ZA-LDH and ZA had a prominent layered shape; however, they differ in their size by about 40 nm. The levodopa intercalated nanocomposite before and after Tween-80 showed particulate type layered shape with almost similar particle size distribution. Details of the sizes, shape and average distribution were presented in Figure 7 and Table 6.

**Table 6:** **Particle sizes**, **shape and distribution pattern**

**Sample**	**ZA** **-** **LDH**	**ZAL** **-** **LDH**	**ZAL**	**ZA**
Shape	Layered type	Particulate type layered	Layered type	Very narrow layered particulate type
Average size	55 nm	32 nm	31 nm	97 nm
Particle size distribution	Narrow	Narrow	Narrow	Narrow

Table showing particle sizes, shape and distribution for both intercalated and un-intercalated zinc-aluminium nanocomposite before and after coating with Tween-80. ZA-LDH (zinc-aluminium layered double hydroxide nanocomposite), ZAL-LDH (zinc-aluminium-levodopa layered double hydroxide nanocomposite), ZAL (zinc-aluminium-levodopa layered double hydroxide nanocomposite coated with Tween-80), ZA (zinc-aluminium layered double hydroxide nanocomposite coated with Tween-80).

### Biodistribution studies

**Figure 8:**
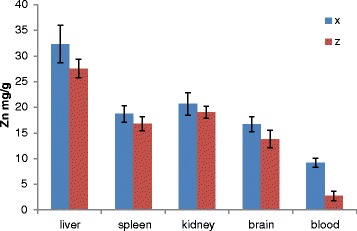
**Graph showing nanocomposite distribution in tissues of rats 14 days after single oral dose administration.**. Zinc distribution in rats after single dose of Tween-80-coated zinc-aluminium-levodopa layered double hydroxide nanocomposite (ZAL), tissue collected 14 days after treatment with 2,000 mg/kg (X) and 100 mL/kg of PBS (Z). No significant difference found between control and treated group in any of tissues analysed *P* > 0.05.

**Figure 9:**
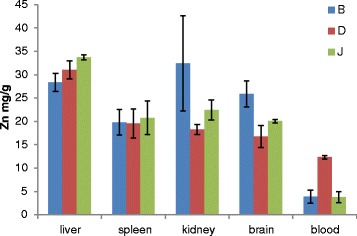
**Graph showing nanocomposite distribution after repeated doses in a 28**-**day oral toxicity study.** Graph showing nanocomposite distribution in tissues of rats after repeated doses in a 28-day oral toxicity study. Zinc distribution in rats after repeated dose of Tween-80-coated zinc-aluminium-levodopa layered double hydroxide nanocomposite ( ZAL) over 28-day period. *B* = 500 mg/kg of ZAL, *D* = 5 mg/kg of ZAL, *J* = 100 mL/kg of PBS (control group). No significant difference found between control and treated group in any of tissues analysed *P* > 0.05.

The Tween-80-coated nanocomposite showed a wider distribution after oral administration from both repeated and single-dose application, respectively (Figures 8 and 9). Reasonable amount of zinc element was found in the liver, kidney, blood and brain of the treated rats compared to the control.

## Discussion

The term ‘nano-medicine’, is a branch of nano-biotechnology where nano-scale devices are used to diagnose and or cure diseases [26]. These devices are generally characterized with site-specific therapeutic action and high potential in curtailing side effects [26]. However, achieving these objectives required a well-designed study, so that the beneficial impact of nano-medicine is not hampered by incomplete information, especially on the safety aspect of the drug.

In acute toxicity studies, animal mortality could be explained via simple observation of clinical signs followed by necropsy [27]. Toxicity signs like a convulsion, abnormal posture, paralysis followed by abnormal necropsy findings on the brain may be pointing to targeted brain toxicity. Where mortality followed these symptoms/signs, a probable cause of death will be established [27]. In this study, ZAL and ZA nanocomposite with and without intercalated levodopa, respectively, were tested at a limit dose based on OECD guidelines 420 [24]. However, no animals exhibited any sign of clinical toxicity; the treatment did not alter their feeding or water intake. This indicated that the newly synthesized LDH-levodopa nanocomposite may be categorized as nanodelivery materials with no obvious clinical toxicity when administered orally for a short period. More than three decades ago, Clarke and Clarke described substances are to be safe and or of low toxicity if they have LD50 of 1,000 mg/kg body weight after an oral route administration [28].

Variation in body weight, especially when control group is compared to treatment groups during toxicological study, can be one of the many indicators of the substance toxicity [29]. However, rats from ZAL, ZA and VC group continued to gain weight over the 2-week study period (Figure 1). In clinical settings, weight loss, in excess of 10% of the initial body weight, is considered scientifically significant [30]. Among the various causes leading to weight loss in either acute or chronic toxicity are direct decrease in food/water intakes or secondary damages to major organs such as the stomach, intestine, liver and kidney by the substance in question, thus altering the normal physiology [29,31]. Recently, layered double hydroxide of magnesium-aluminium was administered to male Balb/c mice at four different doses [32]. The changes in the body weight pattern, demonstrated following a single-dose oral administration of the LDH nanoparticles in these mice, were found to be similar to the findings presented in this study.

Another index of toxicity is the individual internal organ weight in relation to whole animal body weight, which we assessed through the coefficient of the liver, kidneys, brain and spleen in this study. It is simple but sensitive in assessing toxic substance exposure to the animals. As shown in Table 4, no significant changes were observed in the liver, kidney, spleen and brain of ZAL and ZA group compared to VC group. Similar results were presented in another study, where the organosomatic indices of the brain, heart, intestine, kidney, liver, lung, spleen and stomach from mice exposed to LDH nanoparticle via the oral route were remarkably unaffected even at 2,000 mg/kg of the nanoparticle concentration [32]. However, in a related study, an inorganic nanodelivery system of zinc oxide was shown to cause weight loss in the heart, lung, kidneys, spleen, liver and pancreas after oral administration [29]. The same nanoparticle caused an increase in the weight of kidney, spleen and pancreas after intraperitoneal administration. However, it is postulated that the toxicity potential of the nanodelivery system could be based on route of administration.

In the presence of liver damage due to drug toxicity or other diseases, enzymes like alanine aminotransferase (ALT), aspartate aminotransferase (AST), alkaline phosphatase (ALP) and gamma-glutamyl transpeptidase (GGT) are usually elevated [33]. Where there is a twofold or more of these enzymes increments, it is considered significant [33]. The enzymes AST and ALT are elevated either due to liver cell inflammation and subsequent necrosis or as a result of bile flow obstruction causing elevation in GGT, ALP or both [34]. There is abandonment of macrophages in the liver, spleen and bone marrow, allowing for sequestration of nanoparticle in these organs, thus making them more vulnerable to its toxicity [34]. Previously, studies showed liver cell damage with elevated ALT and AST, following oral administration of salts containing zinc and zinc oxide nanoparticles [35,36]. The vast role played by the liver in drug metabolism and the tendency for nanoparticle sequestration in the liver [37] necessitates assessment of the liver functions in this study. The liver enzymes from ZAL- and ZA-treated rats were found to be almost similar to those of the VC (control) group. AST from ZAL (152 ± 30) was shown to be slightly elevated compared to what was obtained from ZA (111 ± 18) or VC (114 ± 11) (Table 4). In hepatocellular injury or necrosis, ALT and AST are the two crucial enzymes that may be raised [38]; but of the two enzymes, ALT is more specific and significant. This is because of its existence mainly in the cytoplasm of liver cells, while AST has both mitochondrial and cytosol type. It also exists in other tissues like the heart, skeletal muscle, brain, liver, pancreas, kidneys, lungs and white and red blood cells [38]. The slight differences noted were found to be statistically insignificant (*P* > 0.05). The ratio of AST to ALT (Table 5) in the treated groups was statistically not different from control. Assessing AST/ALT ratio can further give clues to what causes liver damage where it occurs [39]. Thus, oral ingestion of ZAL and ZA at 2,000 mg/kg in Sprague Dawley rats did not induce any significant liver damage biochemically.

Damage to the kidneys or dehydration can be detected through blood biochemical analysis, looking at urea, electrolytes and creatinine in order to assess changes to the renal system. There was no elevation in serum urea, electrolytes (Na^+^, K^+^, Cl^−^) or creatinine in the two treated groups as compared to the vehicle control group. Statistically, there is no any significant difference between the groups (Table 4). Body exposure to noxious drugs as well as other toxins can easily lead to nephrotoxicity, usually treatable and reversible, but can progress to a permanent renal damage and its consequent outcome [40]. None of these changes associated with renal impairment were observed after 2,000 mg/kg of the ZAL and ZA nanocomposite were given to the rats via the oral route. The normal urea and electrolyte results further attested to the said regular water intake of the treated and control groups, which was observed within the observation period. This is because severe dehydration will alter the urea and some electrolyte levels, notably Na^+^ and K^+^ [41]. Movement of water and electrolyte inside and outside the cells will be deranged as well [41]. Nephrotoxicity was reported in 5 mg/kg of titanium dioxide nanoparticles of different sizes administered to animals via the oral route [37]. In this study, 2,000 mg/kg ZAL and ZA administered to the rats did not cause any change in water intake nor do they damage the renal system of the animals.

On gross examination of internal organs of rats from ZAL, ZA and VC groups, no lesions or anomalies were found. This complemented the increase in weight, absence of morbidity, mortality and excellent clinical status that were generally noted during animal observation (Tables 3 and 4).

Zinc distribution in the treated and untreated animals of both single and repeated dose study groups follows the reported normal pattern of zinc distribution in the body [42]. The liver was shown to accumulate more zinc ion than the spleen, kidney, brain and blood. The distribution of zinc in Figure 8 is showing higher concentration in the liver of the treated rats than the control, also higher concentration in the kidney, spleen, brain and blood samples of the treated rats than their control counterparts. Figure 9 which is the distribution analysis from the repeated dose study showed higher concentrations of the zinc ion in the blood, brain and kidney of the treated rats compared to the control group. The spleen and the liver on the contrary demonstrated slightly lower concentration in the treated samples compared to the control. In the past, inductile couple plasma (ICP) was used to analyse the concentration Mg metal after intravenous injection of anti-cancer LDH nanocomposite to a group of mice [43]. There, liver tissue shows consistent higher concentration of the metal compared to other organs tested; the kidney on the other hand showed increase concentration of Mg metal with increasing time, likely as a result renal excretion after circulation. The concentration of zinc in the kidneys of treated rats in this study is higher than the control group. This is more so in the 500 mg/kg repeated-dose treated group attributable to higher concentration used in the treated group. The concentration is higher than the control but less than that seen in the single-dose study as the samples were collected 14 days after the treatment. Interestingly, the blood sample of the nanocomposite-treated rats demonstrated zinc metal accumulation more so in the 5 mg/kg repeated-dose group, collected about 24 h after the last dose. The indication here is the wider distribution of the LDH nanocomposite after oral administration of different concentrations and the delivery affecting many organs including the brain. Two things likely responsible for this wider distribution and even delivery of this nanocomposite to the brain are the particle sizes (55 nm) and the surface coating with Tween-80.

## Conclusion

In this study, the acute toxicity potential of zinc-aluminium nanocomposite intercalated with levodopa (ZAL) via oral route at 2,000 mg/kg is evaluated in whole animals. Neither the intercalated hybrid (ZAL) nor the un-intercalated nano-hybrid showed any clinical, biochemical or pathological perturbation after 14 days of treatment. The AST/ALT ration of the two treated groups is not significantly different from control. The distribution of the nanocomposite after single and repeated oral doses covered most of the body organs including the brain. However, there is the need for further toxicity study, emphasizing on chronic and gene toxicity assessment and focusing more on possible liver and renal damage.

## Abbreviations

BBB: blood brain barrier
LDH: layered double hydroxide
XRD: X-ray diffraction
FTIR: Fourier transform infrared spectroscopy
Tween-80: polysorbate-80
IACUC: Institutional Animal Care and Use Committee
OECD: Organization for Economic Co-operation and Development
ZAL: zinc-aluminium-levodopa
ZA: zinc-aluminium nanocomposite
PFA: paraformaldehyde
H&E: haematoxylin-eosin
SD: standard deviations
ANOVA: one-way analysis of variance
LD50: lethal dose
AST: aspartate aminotransferase
ALT: alanine aminotransferase
CK: creatine kinase
Creat: creatinine
GGT: gamma-glutamyl transferase
Na+: sodium
K+: potassium
Cl−: chloride
PT: portal triad
CV: central vein
H: hepatocytes
S: sinusoid
WP: white pulp
RP: red pulp
G: glomerular
T: tubule
